# *In Vivo* Articular Cartilage Regeneration Using Human Dental Pulp Stem Cells Cultured in an Alginate Scaffold: A Preliminary Study

**DOI:** 10.1155/2017/8309256

**Published:** 2017-08-16

**Authors:** Manuel Mata, Lara Milian, Maria Oliver, Javier Zurriaga, Maria Sancho-Tello, Jose Javier Martin de Llano, Carmen Carda

**Affiliations:** ^1^Department of Pathology, Faculty of Medicine and Odontology, University of Valencia, Blasco Ibáñez Avenue 15, 46010 Valencia, Spain; ^2^Centro de Investigación Biomédica en Red de Enfermedades Respiratorias (CIBERES), Monforte de Lemos Avenue, 3-5, Pabellón 11, 28029 Madrid, Spain; ^3^Fundación para la Investigación del Hospital Clínico de la Comunidad Valenciana (INCLIVA), Blasco Ibañez Avenue 15, 46010 Valencia, Spain; ^4^Hospital Clinico Universitario de Valencia, Blasco Ibañez Avenue 17, 46010 Valencia, Spain; ^5^Centro de Investigación Biomédica en Red en Bioingeniería Biomateriales y Nanomedicina (CIBERBBN), Monforte de Lemos Avenue, 3-5, Pabellón 11, 28029 Madrid, Spain

## Abstract

Osteoarthritis is an inflammatory disease in which all joint-related elements, articular cartilage in particular, are affected. The poor regeneration capacity of this tissue together with the lack of pharmacological treatment has led to the development of regenerative medicine methodologies including microfracture and autologous chondrocyte implantation (ACI). The effectiveness of ACI has been shown *in vitro* and *in vivo*, but the use of other cell types, including bone marrow and adipose-derived mesenchymal stem cells, is necessary because of the poor proliferation rate of isolated articular chondrocytes. In this investigation, we assessed the chondrogenic ability of human dental pulp stem cells (hDPSCs) to regenerate cartilage *in vitro* and *in vivo*. hDPSCs and primary isolated rabbit chondrocytes were cultured in chondrogenic culture medium and found to express collagen II and aggrecan. Both cell types were cultured in 3% alginate hydrogels and implanted in a rabbit model of cartilage damage. Three months after surgery, significant cartilage regeneration was observed, particularly in the animals implanted with hDPSCs. Although the results presented here are preliminary, they suggest that hDPSCs may be useful for regeneration of articular cartilage.

## 1. Introduction

Osteoarthritis (OA) is a complex systemic disease in which the whole joint, including the synovium, articular cartilage, subchondral bone, tendons, and muscles, is affected [1]. OA can be idiopathic or initiated by aging, trauma, malformations, or inflammatory disease [2, 3]. As it affects up to 10% of males and 18% of females greater than 48 yr of age, OA is becoming a serious health problem, and currently established therapies for OA insufficiently address the clinical need [1].

The loss of articular cartilage distinctive of OA is characterized by proteolytic degradation of the chondral matrix, which induces the release of cytokines including interleukin 6 (IL-6), IL-1*β*, and tumor necrosis factor alpha (TNF-*α*), leading to secretion of matrix-degrading enzymes from chondrocytes, further propagating tissue breakdown [4, 5]. The limited regeneration capacity of articular cartilage generally leads to formation of fibrocartilage-like scar tissue, which replaces the native hyaline cartilage matrix [6].

Current pharmacological agents for the treatment of OA involve anti-inflammatory drugs including cyclooxygenase 2-selective or nonselective nonsteroidal anti-inflammatory drugs as well as TNF-*α*-binding antibodies or IL-1 inhibitors [7–9]. However, the potential for these compounds to improve the structural damage is limited, and the development of novel immune modulation strategies will be necessary to alter the progression of OA [6].

Nonpharmacological regenerative techniques have been developed for regeneration of articular cartilage according to the 3R paradigm: reconstruction, repair, and replacement [10]. Microfracture as well as autogenic (mosaicplasty) and allogenic tissue transplantation has shown positive results in short term but poor outcomes in long term, resulting in poor hyaline cartilage regeneration that is replaced by fibrocartilage [11, 12]. Autologous chondrocyte implantation has shown better results in terms of regeneration not only in experimental studies but also in clinical trials in which this methodology has been compared with microfracture [13].

The principal limitations associated with the use of autologous cartilage include the poor proliferation of autologous chondrocytes as well as the source limitation and morbidity of the extraction process. It has been previously shown that a higher cellular content induces better tissue repair [14, 15]. Native articular cartilage has a cell density of 1.4 × 10^7^/cm, corresponding to 5–10% of the cartilage volume, but this cell density is difficult to replicate *in vitro* [16]. For this reason, other sources for cartilage repair have been evaluated, including bone marrow mesenchymal stem cells (MSCs) or adipose stem cells [17, 18], which have been considered top candidates for cartilage regeneration because of their ability to generate functional cartilage tissue [11].

Human dental pulp stem cells (hDPSCs) are self-renewing MSCs residing within the perivascular niche of the dental pulp [19–21] thought to originate from the cranial neural crest and express both MSC and neural stem cell markers [22]. hDPSCs are easily obtained from extracted third molars, and under specific conditions, they can differentiate *in vitro* into a variety of cell types including neurons, odontoblasts, osteoblasts, adipocytes, and chondrocytes [23, 24]. Nevertheless, their ability to regenerate articular cartilage *in vivo* is poorly understood.

The culture environment dramatically influences chondrogenesis. It has been shown that the three-dimensional environment provided by hydrogels improves cartilage formation [11]. Several hydrogel sources, including proteins such as collagen, elastin, fibrin, and silk fibroin; polysaccharides such as chitosan, chondroitin sulfate, and hyaluronic acid; and seaweed polysaccharides such as alginate, agarose, carrageenan, and ulvan, have been used for cartilage repair [11]. Algal polysaccharides such as alginate have been considered for cartilage regeneration because of their sulfate groups, chemical affinity for mammalian glycosaminoglycans, and lack of interaction with cell integrins that help retain the rounded shape of cultured cells, enhancing chondrogenesis [25, 26].

The objective of this study was to assess the effectiveness of hDPSCs in the regeneration of articular cartilage *in vivo*. hDPSCs and rabbit articular chondrocytes were isolated, characterized, and cultured in a 3% alginate scaffold, and the scaffolds were implanted in an experimental rabbit model of a full-depth chondral joint defect. The results presented here demonstrate the chondrogenic capacity of hDPSCs and suggest their use in cartilage regeneration.

## 2. Materials and Methods

### 2.1. Cell Culture and Experimental Design

Rabbit primary articular chondrocytes and hDPSCs were used in this study. Chondrocytes were isolated as described previously [27]. Briefly, articular cartilage was obtained from the knee joints of donor rabbits following sacrifice by a lethal intravenous injection of anesthetic into the auricular vein (500 mg/iv sodium thiopental; thiobarbital, B. Braun Medical, Barcelona, Spain). The cartilage was dissected from the subchondral bone, finely diced, and washed with Dulbecco's modified Eagle's medium (DMEM; Thermo Fisher Scientific, Fife, WA, USA) supplemented with 100 U penicillin, 100 *μ*g streptomycin (Biological Industries, Kibbutz Beit HaEmek, Israel), and 0.4% fungizone (Gibco/Thermo Fisher Scientific). The diced cartilage was digested with different enzymes in the supplemented DMEM. The cartilage was first incubated with 0.5 mg/mL hyaluronidase (Sigma-Aldrich, St. Louis, MO, USA) in a shaking water bath at 37°C for 30 min. The hyaluronidase was removed, and 1 mg/mL pronase (VWR International, Barcelona, Spain) was added. After incubation in a shaking water bath at 37°C for 60 min, the cartilage pieces were washed with supplemented DMEM. The medium was removed, 0.5 mg/mL collagenase-IA (Sigma-Aldrich) was added, and digestion was continued overnight in a shaking water bath at 37°C. The resulting cell suspension was filtered through a 70 *μ*m-pore nylon filter (BD Biosciences, San Jose, CA, USA) to remove tissue debris. Cells were centrifuged and washed with DMEM supplemented with 10% fetal bovine serum (FBS; Thermo Fisher Scientific). Finally, isolated cells were used immediately for chondrocyte culture by placing them in T75 culture flasks (Becton-Dickinson, East Rutherford, NJ, USA) at a high density in DMEM supplemented with 10% FBS and 50 *μ*g/mL ascorbic acid (Sigma-Aldrich) at 37°C in a 5% CO_2_-humidified atmosphere.

hDPSCs were isolated as described previously [28]. Donors provided informed consent. The study was conducted in accordance with the Declaration of Helsinki and applicable local regulatory requirements and laws. All procedures were approved by the Ethics Committee of the University Clinical Hospital of Valencia (Spain). The dental pulp of human third molars was gently removed under sterile conditions using cow horn forceps with a small excavator and immersed in culture tubes filled with medium. The specimens were then divided into small pieces using a bistoury knife, immersed in Hanks solution (Gibco), and incubated for 2 h at 37°C in 5% CO_2_. The supernatant medium was removed, and 0.1% type IV collagenase (Sigma-Aldrich) was added for 15 min, followed by centrifugation at 1500 rpm for 10 min. The supernatant was removed, and the cells were plated in 25 cm^3^ flasks in DMEM (Nunc, Sigma-Aldrich, Madrid, Spain) containing penicillin/streptomycin, 10% FBS (Sigma-Aldrich, Madrid, Spain), amphotericin B, and 0.1% L-glutamine. The medium was replaced every 4 d. Once the cells reached confluence, flow cytometry was performed.

Rabbit chondrocytes and hDPSCs were cultured in chondral differentiation medium, which was composed of DMEM with 1% insulin-transferrin-sodium selenite medium supplement (BD Biosciences, Madrid, Spain) and 50 *μ*g/mL ascorbic acid for up to 6 wk. Chondrocyte differentiation was evaluated by immunofluorescence using specific antibodies against collagens I (COLI) and II (COLII) and aggrecan (ACAN). Changes in cell morphology were evaluated by fluorescence using rhodamine-conjugated phalloidin.

For *in vivo* experiments, 3% alginate hydrogels containing nondifferentiated primary chondrocytes or hDPSCs were constructed and implanted in rabbit knees. The following experimental groups were evaluated: control (alginate only), alginate containing chondrocytes, and alginate-containing hDPSCs. Three months after surgery, the animals were sacrificed, and a histopathological study of the knees was performed using the contralateral knee of each animal as a control. Three different animals were used in each experimental group.

### 2.2. Flow Cytometric Characterization of hDPSCs

hDPSCs were characterized using a FACSCalibur equipped with a 488 nm Argon laser and a 635 nm red diode laser (Becton Dickinson, Madrid, Spain) as described previously [29]. Experimental data were analyzed using CellQuest software (Becton Dickinson, Madrid, Spain). To exclude debris, samples were gated based on light-scattering properties in the side-scattered and forward-scattered light modes, and 10,000 events per sample within this gate (R1) were collected, using the medium setting for the sample flow rate. The following markers were evaluated: CD29 (Alexa Fluor® 488), CD31 (PE/Cy7), CD44 (PE/Cy5), CD45 (Pacific Blue™), CD105 (APC), and CD146 (PE).

### 2.3. Immunofluorescence Staining of Collagen I, Collagen II, and Aggrecan

Expression of COLI, COLII, and ACAN was determined in cell culture using specific antibodies (Sigma-Aldrich, Madrid, Spain). The cells were cultured on poly-L-lysine-coated cover slides in chondrogenic culture medium for up to 6 wk as described above. Then, they were fixed with 4% paraformaldehyde in phosphate-buffered saline (PBS) pH 7.4 for 10 min. Once washed, the cells were permeabilized with 0.1% Triton X-100 in PBS for 5 min, and after three washes, they were incubated for 30 min with blocking solution (1% bovine serum albumin (BSA) and 1.1% Tween-20 in PBS). The cells were then incubated with the appropriate primary antibody (diluted in antibody diluent at 1 : 100 for COLI and ACAN and 1 : 500 for COLII) overnight at 4°C. After three washes, cells were incubated with 1 : 200 secondary anti-mouse (COLI and ACAN) or anti-rabbit (COLII) FITC-conjugated antibody (Sigma-Aldrich, Madrid, Spain). After the final washes, the nuclei were stained with 4′,6-diamidino-2-phenylindole (DAPI), and the samples were analyzed using the Leica DM2500 fluorescence microscope (Leica, Wetzlar, Germany).

### 2.4. Fluorescence Staining of F-Actin

F-Actin was evaluated using rhodamine-conjugated phalloidin (Molecular Probes, Thermo Fisher Scientific). Cells were cultured on cover slides, grown to subconfluence, washed with PBS pH 7.4, and fixed in 3.7% formaldehyde solution in PBS for 10 min at room temperature. The cells were permeabilized with 0.1% Triton X-100 in PBS for 3–5 min. To reduce nonspecific background staining, samples were preincubated with PBS containing 1% BSA for 20–30 min. Next, each sample was stained for 20 min with 5 *μ*L phalloidin methanol stock solution diluted in 200 *μ*L PBS. Finally, after washing the samples, the nuclei were stained with DAPI and analyzed using the Leica DM2500 fluorescence microscope.

### 2.5. Preparation of Alginate Hydrogels

To prepare alginate scaffolds, a 3% alginate solution was prepared in sterile PBS (Sigma-Aldrich, Madrid, Spain). The alginate solution was filtered through a sterile 0.2 *μ*M syringe filter and stored at 4°C until use. To polymerize the alginate, 50 *μ*L 0.5 M CaCl_2_ prepared in water was added to the bottom of a 24-well culture plate. The alginate solution was warmed at 37°C, and 500 *μ*L was added to the CaCl_2_ solution. The plate was incubated for 30 min at room temperature and then for another 30 min at 4°C. The polymerized scaffold was then covered with 500 *μ*L culture medium and incubated at 37°C in 5% CO_2_. In the experimental groups containing cells, a suspension of 2 × 10^6^ cells/mL in prewarmed alginate solution was prepared, and polymerization was performed as described above.

### 2.6. Scaffold Implantation of Animals

Two-month-old male New Zealand rabbits, weighing 1.5–2.0 kg, were obtained from Granjas San Bernardo S.L. (Tulebra, Spain), quarantined for 7 d, and maintained under conventional housing conditions, with appropriate bedding and free access to water and food. Rabbits were kept in standard single cages under controlled temperature and light conditions.

Spanish guidelines for the care and use of laboratory animals were observed. The study protocol was approved by the Ethics Committee of the University of Valencia according to law 86/609/EEC and 214/1997 and decree 164/1998 of the Generalitat Valenciana Government.

**Figure 1:**
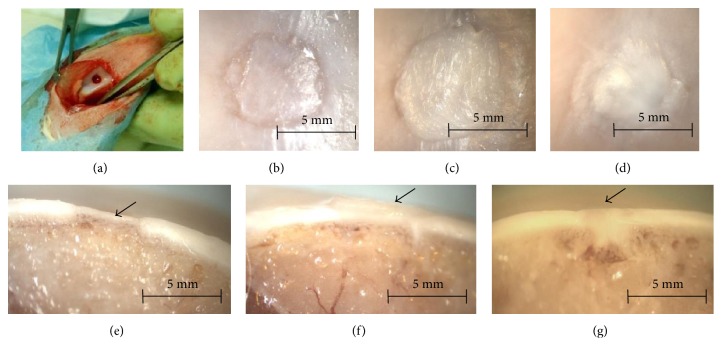
Osteochondral defects of 3 mm were generated in the knees of rabbits (a) and covered with alginate alone (b, e), alginate containing 2 × 10^6^ rabbit primary chondrocytes (c, f), or alginate containing 2 × 10^6^ hDPSCs (d, g). The animals were sacrificed 3 months after surgery. Representative macroscopic images of the animals (*n* = 3) are shown.

Rabbits were preanesthetized by subcutaneous injection of 15 mg/kg ketamine (ketolar; Pfizer, Madrid, Spain) and intramuscular injection of 0.1 mg/kg medetomidine (Domitor; Pfizer) and prepared for surgery (washed, shaved, etc.). General anesthesia was induced with 4% isoflurane using a specially designed mask and maintained by administration of 1.5% isoflurane with O_2_ (2 L/min). The surgical site was sterilized with iodine solution, and nonsterile areas were covered with sterile drapes. Surgeons wore sterile coats and gloves, and all instruments were sterilized beforehand and kept sterile during surgery. An arthrotomy at the knee joint was performed through a medial longitudinal parapatellar incision. The medial capsule was incised and the patella laterally dislocated. A 3 mm steel trephine was used to create a defect of 3 mm in diameter and 1 mm deep in the central articular surface of the femoral trochlear groove, which resulted in subchondral bone injury and removal of articular cartilage (Figure 1(a)). The defect was cleaned and rinsed with sterile saline, and scaffolds were laid in the defect and aligned with the surrounding articular surface. Control animals were subjected to the same operation, but no scaffold was implanted in the cartilage defect. The arthrotomy and skin were sutured with continuous stitches of 4/0 Coated Vicryl (Johnson and Johnson, New Brunswick, NJ, USA). Macroscopic pictures were taken throughout the surgical procedure using a Leica DC150 camera. After removal of the conformed anesthesia mask, rabbits were returned to their cages and allowed free activity. Postoperative analgesia consisted of intramuscular injection of 3 mg/kg dexketoprofen (Enantyum; Menarini, Florence, Italy) on the day of surgery, followed by the same dose every 24 h for 3 d. Three months after surgery, intramuscular injection of 3 mg/kg gentamicin (Genta-Gobens; Laboratorios Normon, Madrid, Spain) was administered as antibiotic prophylaxis. This time point was chosen based on previous studies using the same animal model [27].

### 2.7. Histological Studies

Morphology was evaluated following standard histological procedures. Briefly, rabbit articulation specimens were rinsed with PBS and fixed with 4% formaldehyde at room temperature for 5 d. Samples were rinsed with PBS and immersed in OSTEOSOFT decalcifier solution (Merck, Whitehouse Station, NJ, USA) for 5 wk at room temperature. Specimens were cut through the middle of the scaffold, the diameter of which measured approximately 3 mm, and each half was embedded in paraffin separately, generating 5 *μ*m thick sections, which were stained with hematoxylin and eosin. Stained sections were analyzed under an optical microscope (DM 4000B; Leica) and photographed using the Leica DFC 420 camera. Collagen fiber orientation was analyzed under polarized light using an optical microscope (DM 4000B; Leica) as described previously [12].

Type I and II collagen expression were evaluated by immunohistochemistry using specific mouse anti-human antibodies (C2456 Sigma, dilution 1 : 150 and CP18 Calbiochem, dilution 1 : 100). Sections were deparaffined and rehydrated through graded ethanol, rinsed in distilled water, and treated with 0.3% H_2_O_2_ and 10% normal horse serum to block endogenous peroxidase and nonspecific binding, respectively. Antigen retrieval for type I and type II collagen was performed by proteinase k incubation for 10 min. Dako envision amplification system (Cytomation Envision System-labelled polymer-HRP anti-mouse) was used, followed by development with 3,3′-diaminobenzidine (Dako, Barcelona, Spain) as chromogen according to the manufacturer's instructions, which originated a brown staining in immunoreactive structures. Sections were finally counterstained with Mayer's hematoxylin (Sigma-Aldrich, Madrid, Spain).

### 2.8. Data Presentation

All experiments were performed in triplicate. The histopathological study was performed in a double-blinded manner, and the figures presented in the manuscript are representative images.

## 3. Results

### 3.1. Differentiation of Rabbit Primary Chondrocytes *In Vitro*

**Figure 2:**
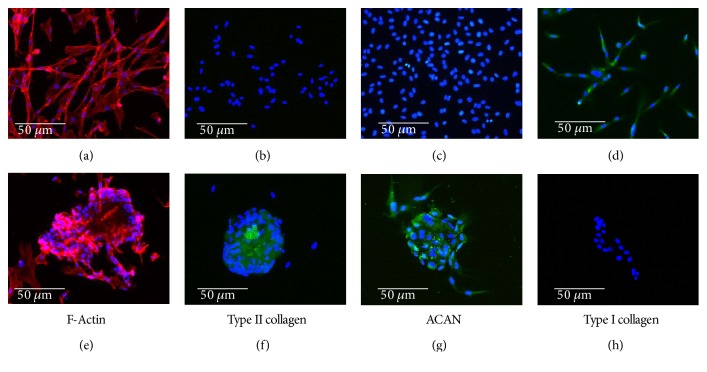
Rabbit primary articular chondrocytes were isolated and cultured in proliferation (a–d) or differentiation culture medium (e–h) for up to 6 weeks. F-Actin expression (a, e) was analyzed by microscopy fluorescence using rhodamine-conjugated phalloidin, and collagen II (COLII; (b, f)), aggrecan (ACAN; (c, g)), and collagen I (COLI; (d, h)) expression were analyzed by immunofluorescence. In all panels, cell nuclei were stained with 4′,6-diamidino-2-phenylindole (DAPI). Microphotographs are representative fields of three independent experiments.

Rabbit primary chondrocytes were isolated and cultured as described in Materials and Methods. Cells were then cultured in proliferation or differentiation culture medium for up to 6 wk. Cell morphology was evaluated using rhodamine-conjugated phalloidin, while COLI, COLII, and ACAN expression were analyzed by immunofluorescence. The results are summarized in Figure 2. Chondrocytes grown in proliferation medium demonstrated a stellate mesenchymal morphology with marked F-actin stress fibers (Figure 2(a)) as well as expression of type I collagen (Figure 2(d)). The shape of cells cultured in chondral differentiation medium changed dramatically to a rounded morphology (Figure 2(f)), with diminished fibrillary actin. These changes correlated with significant expression of COLII and ACAN (Figures 2(g) and 2(h)). No expression of COLI was detected (Figure 2(i)).

### 3.2. Characterization and Differentiation of hDPSCs *In Vitro*

hDPSCs were cultured and analyzed by flow cytometry for CD29, CD44, CD105, and CD146 expression. Nearly 98% of the cells analyzed were positive for CD29, CD44, CD105, and CD146 but negative for CD31 and CD45.

**Figure 3:**
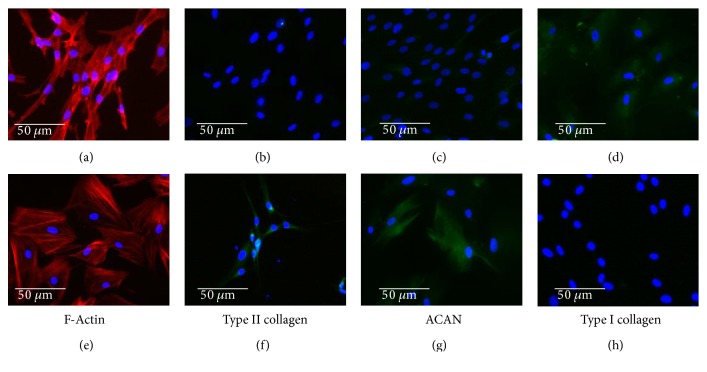
Human dental pulp stem cells (hDPSCs) were isolated and cultured in proliferation (a–d) or chondrocyte differentiation culture medium (e–h) for up to 6 weeks. F-Actin expression (a, e) was analyzed by fluorescence microscopy using rhodamine-conjugated phalloidin, and COLII (b, f), ACAN (c, g), and COLI (d, h) expression were analyzed by immunofluorescence. In all panels, cell nuclei were stained with DAPI. Microphotographs are representative fields of three independent experiments.

The cells were grown in proliferation or differentiation culture medium for up to 6 wk, and the results are summarized in Figure 3. Cells cultured in differentiation medium displayed a more rounded morphology compared with those cultured in proliferation medium. Nevertheless, this change was not as evident as that in primary chondrocytes (Figures 3(a) and 3(e)). No expression of COLI and lower expression of COLII were observed (Figures 3(f) and 3(g)), although significant expression of ACAN was evident (Figure 3(d)).

### 3.3. Chondrocytes and hDPSCs Induce Joint Cartilage Regeneration *In Vivo*

To determine if cultured cells can induce cartilage regeneration, a well-established rabbit model of articular regeneration was used [27]. Alginate scaffolds with or without chondrocytes or hDPSCs at a density of 2 × 10^6^ cells/mL were manufactured and implanted in the knees of rabbits. The animals were sacrificed 3 months after surgery, and the joints were evaluated. Rabbit joints were evaluated macroscopically, and representative results are shown (Figures 1(b), 1(c), 1(d), 1(e), 1(f), and 1(g)). Animals with alginate implants showed a significant loss of articular cartilage with an appreciable sinking of the articular surface (Figures 1(b) and 1(e)). Alginate containing primary chondrocytes resulted in a clear regeneration of articular cartilage, which appeared to protrude into the articular cavity (Figures 1(c) and 1(f), arrow). Similar results were observed with alginate containing hDPSCs, except with less evident protrusion into the joint cavity (Figures 1(d) and 1(g)).

**Figure 4:**
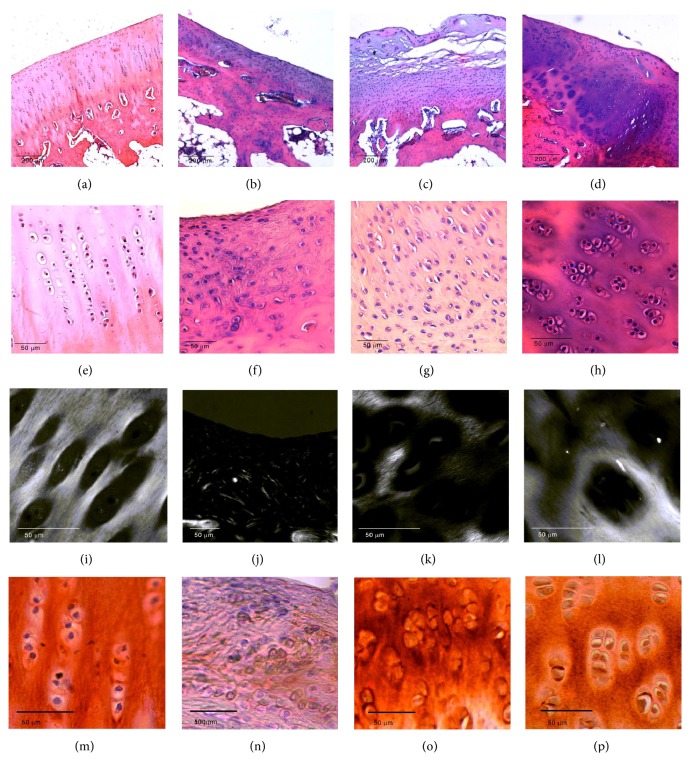
Osteochondral defects of 3 mm were generated in the knees of rabbits and covered with alginate alone (b, f, j, n), alginate containing 2 × 10^6^ rabbit primary chondrocytes (c, g, k, o), or alginate containing 2 × 10^6^ hDPSCs (d, h, l, p). The animals were sacrificed 3 months after surgery. Knees were fixed with 4% formaldehyde, immersed in OSTEOSOFT decalcifier solution for 5 wk at room temperature, and embedded in paraffin. The resulting 5 *μ*m-thick sections were stained with hematoxylin and eosin and analyzed under normal (a–h), polarized (i–l) light using an optical microscope. Immunohistochemistry analysis of type II collagen is represented in panels m–p. Histological images of control animals (a, e, i, m) are also represented. Representative microscopic images of *n* = 3 animals are shown.

Histological analysis revealed a marked loss of articular cartilage in rabbits implanted with alginate only (Figures 4(b) and 4(f)) compared to that in control animals (Figures 4(a) and 4(e)). This loss of cartilage was clearly diminished in animals implanted with alginate containing either chondrocytes (Figures 4(c) and 4(g)) or hDPSCs (Figures 4(d) and 4(h)). These animals exhibited marked regeneration of articular cartilage, characterized by the formation of new isogenic chondral groups and new chondral matrix, although this was more evident in animals implanted with hDPSCs compared with chondrocytes. Polarized light microscopy was used to analyze the arrangement of chondral matrix fibers. A pronounced disturbance in collagen fiber arrangement was observed in the animals implanted with alginate only compared with animals implanted with alginate containing chondrocytes or hDPSCs, in which the fiber disposition was more similar to that of native cartilage (Figures 4(i), 4(j), 4(k), and 4(l)). Immunohistochemistry was used to evaluate type I and II collagen expression. In the animals in which only alginate was used to repair the lesion, a lower expression of type II collagen was observed compared to that in control animals (Figures 4(m) and 4(n)). In both experimental groups in which chondrocytes and hDPSCs were used in combination with alginate, a higher expression of type II collagen was observed (Figures 4(o) and 4(p)). No significant expression of type I collagen was found in the articular cartilage of animals analyzed (data not shown).

## 4. Discussion

OA is considered an inflammatory systemic disease in which all joint components are affected. It is characterized by a progressive degeneration of articular cartilage and nonreversible replacement of this cartilage with nonfunctional fibrocartilage. Articular cartilage is an avascular connective tissue, resulting in poor regenerative capacity [30]. The prevalence of OA increases every year as a consequence of the aging population, and it is considered a serious health problem with no effective pharmacological treatment [6].

According to the International Cartilage Repair Society, microfracture, autologous chondrocyte implantation, and osteochondral autographs result in certain degrees of short-term success for OA treatment [1]. The success of autologous chondrocyte implantation has been demonstrated not only at the experimental level but also in clinical trials, and autologous chondrocyte implantation has shown benefits comparable to those of other options such as microfracture [13]. However, this option suffers from a number of limitations primarily related to the extraction source (cartilage biopsy, which is not exempt from morbidity) and the poor regeneration capacity of chondrocytes, which can make it impossible to obtain the minimum number of cells needed for tissue regeneration [31, 32]. These limitations have stimulated researchers to investigate the use of other cell types. Among them, MSCs of different origin, including bone marrow or adipose, have been evaluated because of their capacity to regenerate cartilage [33].

Recently, dental pulp has gained interest as a source of MSCs. Human dental pulp contains several types of stem cells including hDPSCs, periodontal ligament stem cells, stem cells from apical papilla, and dental follicle progenitor cells [19, 21, 34, 35]. hDPSCs are characterized by a high-proliferative capacity while maintaining the ability to differentiate into multiple lineages [23]. Although these cells have been investigated for their potential to differentiate into chondrocytes *in vitro* [36], their regeneration of cartilage *in vivo* is poorly understood. Here, we found that after growth for 6 wk in chondrocyte differentiation culture medium, hDPSCs express ACAN. Nevertheless, only low expression of COLII was detected. This could be due to the length of time in culture; ACAN is expressed earlier than COLII, so it is possible that an increase in COLII would be seen after longer time points. A second possibility is the culture environment. It has been demonstrated by different groups that a three-dimensional environment is critical for chondrocyte differentiation [11]. Moreover, Nemeth et al. demonstrated that DPSCs form three-dimensional spheroids show significant upregulation of chondrogenic gene markers when cultured in hydrogel scaffolds of composite methacrylated gelatin-hyaluronic acid [37]. Among the different hydrogels used for cartilage regeneration, alginate has been successful for the generation of cartilage [11]. For this reason, we cultured cells in alginate hydrogels to evaluate their ability to regenerate articular cartilage *in vivo.* We used a well-established *in vivo* model of articular cartilage damage [27] and primary isolated chondrocytes as a reference group. Our results demonstrate the importance of using different cells (chondrocytes or mesenchymal cells) to obtain appropriate cartilage regeneration. Animals in which only alginate was implanted demonstrated poor regeneration with loss of cartilage tissue compared with those in which chondrocytes or hDPSCs were also implanted. We observed improved tissue regeneration as well as a smoother articular surface when hDPSCs were used instead of primary chondrocytes, which may be due to the anti-inflammatory effects of hDPSCs (discussed below).

Although the results presented here suggest that hDPSCs are useful for cartilage regeneration, it is important to note that this is a preliminary study. On the one hand, it will be important to further our understanding of the mechanisms involved in hDPSC-induced regeneration. These cells have been shown to inhibit the characteristic inflammatory processes underlying OA [38]. In fact, administration of culture medium conditioned by hDPSCs has confirmed beneficial effects on OA [39]. On the other hand, it will also be important to analyze the composition of the regenerated tissue and to extend this study to other animal models of OA. Despite these limitations, the results shown here are novel and support the use of dental pulp as an accessible source of mesenchymal cells, which may be used instead of primary chondrocytes because of their better proliferation and cartilage-regeneration capacity.
